# Self-Monitoring Performance of 3D-Printed Poly-Ether-Ether-Ketone Carbon Nanotube Composites

**DOI:** 10.3390/polym15010008

**Published:** 2022-12-20

**Authors:** Lorenzo Paleari, Mario Bragaglia, Francesco Fabbrocino, Raimondo Luciano, Francesca Nanni

**Affiliations:** 1 Department of Enterprise Engineering Mario Lucertini, University of Rome Tor Vergata, and INSTM Research Unit Roma Tor Vergata, via del Politecnico 1, 00133 Rome, Italy; 2 Department of Engineering, Pegaso Telematic University, 80143 Naples, Italy; 3 Department of Engineering, University of Naples Partenope, 80133 Naples, Italy

**Keywords:** self-monitoring composites, fused filament fabrication, poly-ether-ether-ketone, carbon nanotubes

## Abstract

In this paper, poly-ether-ether-ketone (PEEK) carbon-nanotube (CNT) self-monitoring composites at different levels of filler loading (i.e., 3, 5 and 10% by weight) have been extruded as 3D-printable filaments, showing gauge factor values of 14.5, 3.36 and 1.99, respectively. CNT composite filaments of 3 and 5 wt% were 3D-printed into tensile samples, while the PEEK 10CNT filament was found to be barely printable. The 3D-printed PEEK 3CNT and PEEK 5CNT composites presented piezo-resistive behavior, with an increase in electrical resistance under mechanical stress, and showed an average gauge factor of 4.46 and 2.03, respectively. Mechanical tests highlighted that 3D-printed samples have a laminate-like behavior, presenting ultimate tensile strength that is always higher than 60 MPa, hence they offer the possibility to detect damages in an orthogonal direction to the applied load wit high sensitivity.

## 1. Introduction

Self-monitoring is the intrinsic ability of a material or structure to detect occurring stress, deformation or damage without the use of external sensors [1,2,3]. Self-monitoring materials are one type of the so-called multifunctional materials, which are characterized by their ability to offer more than a single specific function by means of their specific composition and microstructure. Self-monitoring materials are very important in structural health monitoring (SHM), by which the mechanical integrity of structures or components is checked and assured continuously. A well-performed SHM can bring many beneficial effects in terms of user safety and maintenance costs (as programmed interventions can be scheduled), as well as allowing for the immediate acknowledgment and prevision of residual life when sudden and catastrophic events occur. Traditionally, SHM is achieved through the use of external sensors (strain gauge, fiber optics, accelerometers, etc. [4,5,6]) which, despite offering a high performance, represent an added cost, require cable wiring, are time-consuming during manufacturing, and increase the weight of the final components. The latter feature makes their use a non-environmentally sustainable solution in all applications where a low weight is essential to save fuel/battery charge and to lower CO_2_ emissions (i.e., aerospace, automotive or transport). SHM achieved by using by self-monitoring materials can therefore be an innovative sustainable solution.

In recent years, the advent of additive manufacturing (AM), also known as 3D printing, has drastically boosted the development of new products and technical systems. Freedom in mechanical design, followed by the possibility to manufacture complicated geometries, are the key features of this technique. Among the vast universe of polymer 3D printing, the AM of polymers with superior mechanical, physical and chemical properties, such as so-called high-performance polymers (polyether-ether-ketone PEEK, polyether-ketone-ketone PEKK, polyether-ketone-ether-ketone PEKEK, polyether-imide PEI, etc.), is becoming predominant in many engineering applications in the aerospace, automotive and biomedical sectors [7,8,9,10]. Fused filament fabrication (FFF) is, in most cases, the only 3D-printing technique suitable for the additive manufacturing of these materials, and the recent literature has made it clear that the mechanical properties, performance and failure mode of the printed parts are highly dependent on many process parameters (such as the layout, orientation, infill, parameters, etc.) [11,12]. Moreover, in some cases, despite working with ductile polymers, 3D-printed structures can undergo a more brittle-like, and thus unpredictable, fracture [13]. Therefore, it appears clear how SHM can become important, if not vital, when dealing with 3D-printed polymeric structures. On the other hand, the integration of sensors during the printing job is hard, if not impossible, particularly in the case of high-performing polymers, as they are generally processed at very high temperatures (i.e., >350 °C, which is not sustainable for most electronics and sensors) in closed chambers. The application of sensors after printing is definitely even less efficient. The 3D printing of self-monitoring PEEK is therefore a possible unique solution and appealing target.

Self-monitoring polymeric composite materials are most commonly based on piezo resistivity, i.e., the ability of a material to show a variation of electrical resistivity under strain or damage. For polymers, this property is achieved by manufacturing (nano)composites with specific compositions and microstructures so that a certain degree of electrical conductivity is reached. This is usually conducted through the addition of electrically conductive fillers to the insulating polymeric matrix to reach percolation. The electrical percolation threshold is the critical filler concentration at which the insulator–conductor transition occurs and the conductivity increases sharply by several orders of magnitude due to the formation of a three-dimensional network connected by a contact or tunnelling effect [14]. The percolation threshold value is determined by several parameters, such as the matrix-filler system [15,16,17], filler alignment and distribution [18], filler agglomeration and dispersion [19], as well as processing conditions [20]. Different nanoparticles have been used in the literature to produce electrical conductive 3D-printed thermoplastic-based composites, e.g., carbon black (CB) [21,22], graphene oxide [23], reduced graphene oxide [24], graphene [25,26], carbon fibers [27,28] and carbon nanotubes [29,30]. When a percolating system is deformed, a combination of the deformation of the filler and the modification of the conductive network occurs, and the electrical resistivity of the material changes, giving rise to piezo-resistive behavior [31].

Among different conductive fillers, carbon nanotubes (CNTs) are widely used due to their very high electrical conductivity and low percolation threshold associated with their aspect ratio (ratio of length to diameter) [32]. Particles with a high aspect ratio, indeed, have a higher surface area per unit volume and therefore percolate at a lower content [33]. In CNT-loaded polymer composites, percolation thresholds varying from 0.05 to 5 wt% have been reported in different thermoplastic matrices [34]. The mixing process is also a crucial factor in the determination of the resulting electrical properties. There are several processes to obtain a good dispersion of CNTs, including ultra-sonication with a polymeric dispersant and mechanical mixing (i.e., twin screw mixer, three-roll milling [35].

PEEK–CNT composites produced via various techniques have been reported to present a percolation threshold between 1 and 5 wt% and have been employed as high-performance EMI shielding and electrostatic discharge materials for advanced technological applications [25].

FFF printing of piezo-resistive composites has been sparingly investigated in the literature. The self-monitoring performances of commercial carbon black (CB)-filled acrylonitrile-butadiene-styrene (ABS) filaments were investigated in [36], and the influence of printing parameters has been assessed. ABS, with the addition of 1 to 10 wt% CNT, has been manufactured and 3D-printed, and the sensitivity to an applied deformation has been investigated in static, cyclic and creep testing [37,38]. Multi-material FFF printing to place the responsive material only where needed has been investigated on poly-lactic acid (PLA) and CB systems [39], with flexible polymeric matrices [40,41,42], and ABS–CNT composite testing [43]. A limited amount of research, however, is available on self-monitoring materials based on high-performance 3D-printed polymers, constituting a missed opportunity to employ innovative solutions to SHM in high-performance and weight-critical sectors, such as the aerospace or biomedical sectors. Nanocomposites based on thermoplastic polyurethane (TPU) [44] and thermoplastic polyimide (TPI) [45] have been investigated. Andrew et al. [46] proved that 3D-printed PEEK reinforced with 30 wt% carbon fibers has self-monitoring properties with a gauge factor ranging from 3.1 to 5.2.

In this paper, the preparation of piezo-resistive PEEK filaments and the FFF 3D printing of self-monitoring specimens are described for the first rime. Piezo-resistive PEEK filaments were manufactured by the addition of multiwalled carbon nanotubes (CNTs) to PEEK powder. The self-monitoring behavior of both filaments and 3D-printed parts were assessed, and the mutual influence of mechanical, morphological and thermal properties was investigated.

## 2. Materials and Methods

PEEK–CNT composites were prepared starting from PEEK powder (PEEK 90G, Victrex, Thornton Cleveleys, UK) and carbon nanotubes at different filler loading levels, i.e., 3, 5 and 10 wt%. Commercial multiwall carbon nanotubes were employed (NC7000, Nanocyl, Sambreville, Belgium) with an average diameter of 9.5 nm, length of 1.5 μm and a 250–300 m^2^/g surface area. After dry powder mixing, PEEK–CNT composites were extruded into filaments (1.75 mm diameter) using a single-screw extruder (Filabot series EX2, Filabot, Barre, VT, USA).

**Figure 1:**
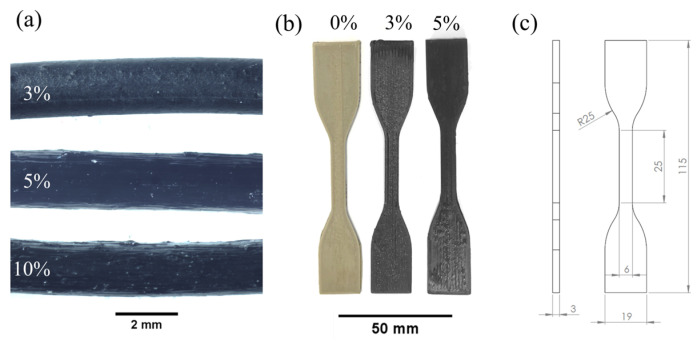
(**a**) Extruded filaments; (**b**) 3D-printed samples; (**c**) tensile specimen dimensions.

The filaments were 3D-printed using an APIUM P155 3D printer (Apium Additive Technologies GmbH, Karlsruhe, Germany) with the following parameters: nozzle temperature 460 °C; build platform temperature, 150 °C; printing speed, 160 mm/min; nozzle diameter, 0.6 mm, layer height, 0.20 mm; and infill percentage, 100%. The 3D models were constructed via SolidWorks (Dassault Systèmes, Vélizy-Villacoublay, France) CAD software, and the G-code was generated using Simplify3D slicing software (Simplify3D, Cincinnati, OH, USA). The composite filaments were 3D-printed into tensile test samples of standard type IV dog-bone geometry according to ASTM D638 [47] (gauge length: 25 mm; overall length: 115 mm; width at narrow section: 6 mm; thickness: 3 mm, see Figure 1c) with a 0° infill raster angle, i.e., all the extruded material parallel to the direction of the applied load during the tensile test.

Electrical percolation was evaluated by measuring the electrical resistivity of 200 mm long filament samples with a digital multimeter (DMM 2700, Keithley, Cleveland, OH, USA) interfaced via software to a PC. Electrical contacts made of copper wires were applied to the specimen ends using a conductive ink (Loctite EDAG 6017SS E&C, Henkel, Dusseldorf, Germany). Volume resistivity was calculated according to Equation (1)

(1) $$\rho =R\frac{A}{L}$$

where *ρ* is the volume resistivity in (Ω·cm), R is measured electrical resistance (Ω), A is the sample cross-section (cm^2^) and L is the distance between electrodes (cm).

The self-monitoring performances of both the filaments and the 3D-printed parts were assessed by measuring the electrical resistance (Keithley DMM 2700 digital multimeter) during the tensile test (Instron 5869, Instron, Norwood, OH, USA, equipped with a 50 kN load cell and tensile grips, crosshead deformation speed of 5%/min). Cyclic tests were performed on 3D-printed samples following a loading–unloading tensile cycling procedure, increasing the amplitude by 5 MPa for each cycle.

Electron scanning microscopy (SEM) micrographs of the gold-sputtered cross-sections of the tensile fractured filaments were acquired using FEG-SEM (Leo supra 35, Zeiss, Wetzlar, Germany). Optical microscopy observations on tensile fractured 3D-printed samples using a stereoscopic zoom microscope (Nikon SMZ-U, Minato, Japan) were performed too.

## 3. Results and Discussion

### 3.1. Electrical Resistivity

Filaments of different compositions (i.e., 0, 3, 5 and 10 wt% CNT) were extruded (Figure 1a) and measured to assess their electrical resistance.

From the electrical resistance measurements, it was found that 3 wt% of CNT produces an average resistivity of 251 Ω·cm (with a sample standard deviation (SSD) of 9 Ω·cm), 5 wt% of CNT produces a resistivity of 16.9 Ω·cm (SSD of 0.8 Ω·cm), and 10 wt% of CNT produces a resistivity of 0.368 Ω·cm (SSD of 0.077 Ω·cm). As expected, the increase in filler content entails a decrease in resistivity due to the formation of a percolating network of particles with decreasing interparticle resistance.

**Figure 2:**
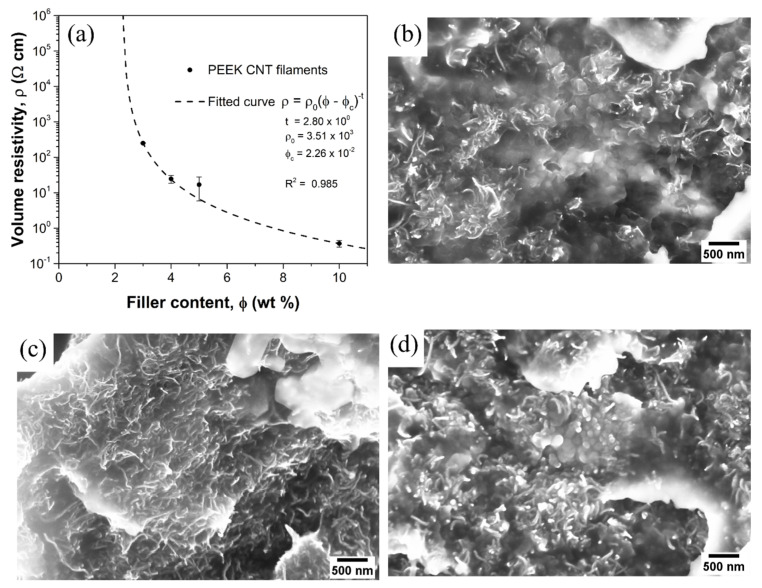
(**a**) Volume resistivity as a function of CNT amount for all filaments; SEM micrographs on tensile tested (**b**) PEEK 3CNT, (**c**) PEEK 5CNT and (**d**) PEEK 10CNT filaments.

Figure 2a displays the measured volume resistivity as a function of filler content. The measured data, with the addition of those coming from 2 wt% CNT and 4 wt% CNT-loaded filaments, prepared in the same way, were fitted using the classic percolation power law in Equation (2) [14]

(2) $$\rho =\rho_{0}\;{\left(\phi -\phi_{c}\right)}^{-t}\;$$

where $\rho_{0}$ is a proportionality constant, ϕ is the mass filler content, $\phi_{c}$ is the percolation threshold and t is the critical exponent. The fitted value for the critical exponent t is 2.80 (with a fitting standard error of 0.33), which is compatible with the literature, for the PEEK–CNT composite systems [48]. The critical exponent is theoretically independent of the specific matrix-filler system and depends only on dimensionality [14], with a theoretical universal value of 1.6–2.0 for three-dimensional systems [48]. Experimentally, however, the distribution of the conductive filler within the insulating matrix gives rise to system-dependent critical exponents [20,49,50] with values up to 3 [51], depending on the filler dimensions, aspect ratio and processing conditions. In the case of needle-like fillers, such as CNT, the percolation threshold is expected to occur at a lower filler content than it would in the case of filler with a different aspect ratio [33,52,53].

The fitting curve shows the percolation threshold $\phi_{c}$ occurs at 2.26 wt% (fitting error of 0.02 wt%), which is compatible with the values reported in the literature for thermoplastic matrices [34,54,55], especially for highly crystalline polymers, such as high-density polyethylene [56] and polypropylene [57]. The formation of the filler network, on the other hand, is influenced by the crystalline regions (PEEK is a high crystalline polymer), as CNTs selectively aggregate and segregate in amorphous regions [58]. They also preferentially distribute in the center of spherulites and crystallites (often promoting nucleation) [59]. Such occurrences have been reported in semi-crystalline polymers, regardless of the type of polymer matrix [59].

All the produced filaments are therefore above the percolation threshold but with different levels of network density and electrical conductivity, which are expected to greatly affect the piezo-resistive behavior of the composites [60].

SEM micrographs on the composite filaments (Figure 2b–d) show the carbon nanotubes appearing randomly and being homogeneously dispersed throughout the PEEK matrix without extensive aggregates. The filler network density, as expected, increases with an increase in filler content, which is the key factor in determining the piezo-resistive sensitivity of the composites. The scarcity of aggregates, granted by the applied shear during the extrusion process which overcomes the CNT-to-CNT van der Waals forces, plays a key role in the determination of the processing, mechanical and electrical properties of the composite. Agglomerates are indeed known to do the following: (i) cause issues during melt processing, i.e., 3D printing; (ii) hinder proper load transfer and act as stress concentrators; and (iii) reduce the number of individual CNTs contributing to the percolation network [61]. Finally, the negligible presence of porosity has been found in the filaments.

### 3.2. Self-Monitoring Performances of PEEK Filaments

**Figure 3:**
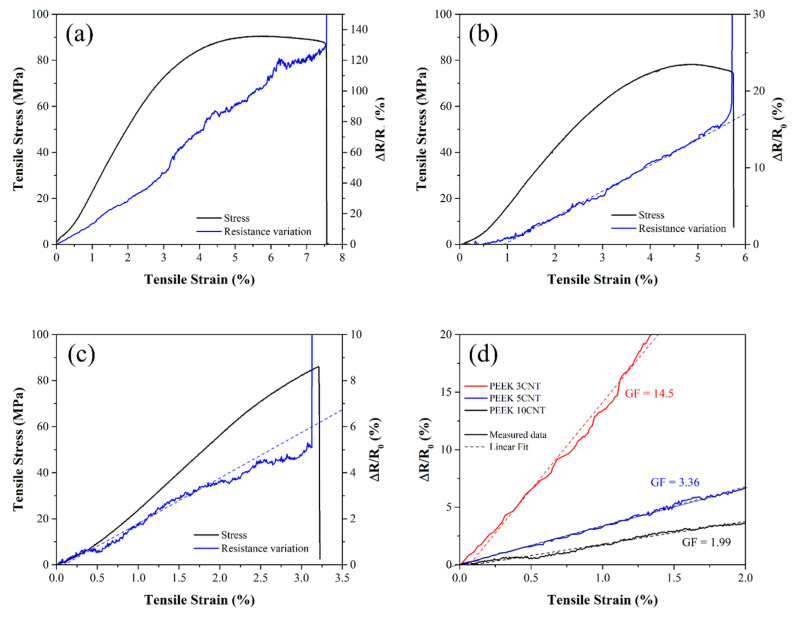
Tensile stress (black line) and electrical resistance (blue line) curves as a function of strain for (**a**) PEEK 3CNT, (**b**) PEEK 5CNT, and (**c**) PEEK 10CNT filament samples and (**d**) comparison of the electrical resistance variation for the filament samples with computed gauge factors.

Figure 3a−c reports the results of the self-monitoring tests carried out on filaments with different CNT loading levels. Each graph reports the stress (σ) vs. strain (ε) curves, as well as the electrical resistance variation vs. strain curves for each type of sample. Electrical resistance variation was calculated as $\;\Delta R/R_{0}=\left(R-R_{0}\right)/R_{0}$, where *R* is the measured resistance during the test and R_0_ is the resistance in the unloaded state. The gauge factor (*GF*) of the system, which can be considered the figure of merit of the self-monitoring performance, is reported in Equation (3) where ε is the mechanical strain.

(3) GF=ΔRR0ε 

All filaments show piezo-resistive behavior, as the electrical resistance increases under increasing strain. Under low strain, the electrical resistance varies almost linearly with the strain in all samples, showing the possibility to linearly correlate the measured electrical resistance with the deformation, giving rise to self-monitoring properties. In Figure 3d, a comparison of the piezo-resistive behavior in the linear elastic region of the three compositions is presented. The 3, 5 and 10 wt% CNT-loaded filaments showed average gauge factors of 14.5, 3.36 and 1.99 (see Table 1), respectively, which are equal to or higher than that of commercial metal strain gauge (GF = 2). All the prepared composites show promising self-monitoring performances in the linear elastic region.

The difference in the GF and sensitivity of the samples can be ascribed to the difference in filler content, which in turn influences the interparticle distance. The interparticle distance in the undeformed system is indeed a crucial factor in the determination of the electrical current flow and, thus, piezo-resistive behavior [62]. The piezo-resistive effect of the CNT-loaded polymers can indeed be related to the following three main factors: (i) the variation in the interparticle distance and the number of CNT–CNT contacts due to filler rotation, reorientation and separation, which lead to an alteration in conductivity; (ii) the variation in tunnelling resistance, namely the resistance in electron-conductivity between non-contacting particles through the tunnelling effect; and (iii) the deformation of the nanotubes, which gives rise to the intrinsic piezo-resistivity of the filler [62,63,64]. While (i) can be considered predominant in the determination of the piezoelectric behavior in systems with an amount of filler above percolation, tunnelling can play a major role when filler concentration is around the percolation threshold. The (iii) mechanism is generally negligible in polymeric composites.

PEEK 3CNT samples were proven to be just above percolation; therefore, they are expected to present a lower density network with few available pathways and a consequent high sensitivity to system alterations, resulting in a very high GF [65]. Denser systems, on the other hand, are characterized by a higher redundancy of pathways; therefore, the applied stress, while affecting the internal distribution of the fillers, does not greatly affect electron flow [66]. This is the case of PEEK 10CNT samples, which are significantly above percolation (very low volume resistivity in the unstressed state) and, coherently, offer a lower gauge factor and self-monitoring sensitivity. As a general remark, it has to be remembered that CNT-based composites always present lower sensitivity than other systems loaded with carbon black or graphene nanoplatelets [60], as the needle-like geometrical shape and the very high aspect ratio of the nanotubes (up to about 1000), while allowing for a lower percolation threshold [33,52,53], causes the particles to maintain contact during deformation through sliding; therefore, they resist separation [67]. Moreover, high aspect ratio particles may be in contact with several other CNTs along its length, thus adding to the redundancy, and form a network with fewer tunnelling contacts compared to lower aspect ratio filler systems (like carbon black).

Above the elastic region, at higher strain levels (i.e., >2%), the electrical resistance variation generally loses linearity and becomes noisy as a result of the irreversible changes in the percolation network caused by PEEK plastic deformation and polymer chains’ reorientation. This is particularly evident in the PEEK 3CNT sample at around 3% strain (Figure 3a). Finally, shortly before mechanical failure, electrical resistance experiences a sudden increase to become infinite when material continuity is interrupted.

### 3.3. Self-Monitoring Performances of 3D-Printed PEEK

The 3 and 5 wt% CNT composite filaments were 3D-printed into tensile samples (Figure 1b) for mechanical and self-monitoring characterization. On the other hand, due to its high filler loading levels, the PEEK 10CNT filament was found to be barely printable, so the quality of the resulting 3D-printed specimens was considered unsatisfactory, and the samples were discarded. The addition of a high amount of CNT, in fact, resulted in a decrease in the flowability of the composite due to the strong particle–particle and filler–matrix interactions [68]. Moreover, PEEK 10CNT filaments were stiff and brittle; therefore, proper feeding, melting and deposition were not possible. The feeding mechanism, indeed, consists of a pinch-roll system in which the solid filament is gripped and pushed through a guiding tube to the heated nozzle. Therefore, the solid filament itself acts as a plunger to extrude the molten polymer through the nozzle. The combination of high viscosity, leading to a higher downstream pressure acting on the filament, and the stiffness and brittleness of the material resulted in the failure of the filament inside the guiding tube due to buckling [69].

**Figure 4:**
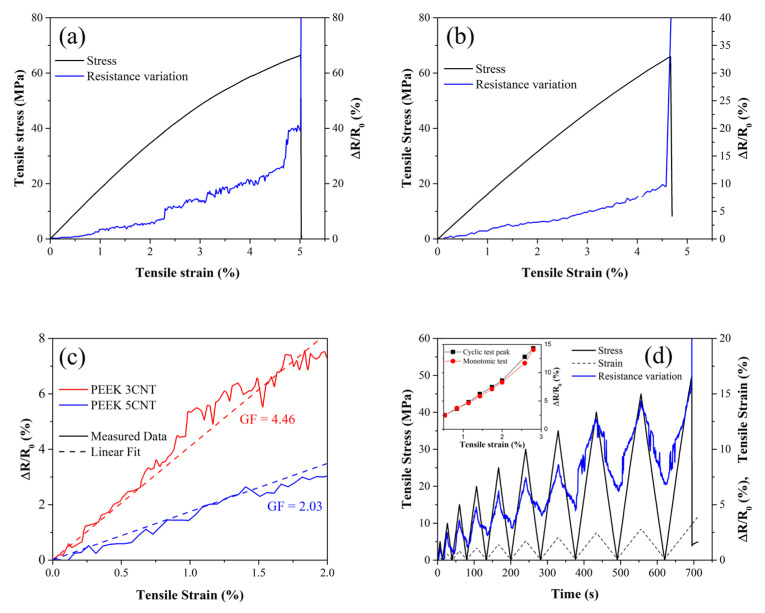
Tensile stress (black line) and electrical resistance (blue line) curves as a function of strain for (**a**) PEEK 3CNT 3D-printed sample and (**b**) PEEK 5CNT 3D-printed sample; (**c**) comparison of the electrical resistance variation with computed gauge factors; (**d**) self-monitoring performances of PEEK 3CNT 3D-printed sample for cyclic loadings; inset: comparison between the maximum values reached in each cycle and the values obtained with monotonic testing.

The results of the self-monitoring characterization of the 3D-printed PEEK 3CNT and PEEK 5CNT samples are reported in Figure 4a,b, while a comparison of the linear region of the curves is presented in Figure 4c,d. The composites present piezo-resistive behavior with an increase in electrical resistance under mechanical stress (i.e., 20.9% and 7.3% resistance increase for PEEK 3CNT and 5CNT, respectively, at a strain of 4%). PEEK 3CNT and PEEK 5CNT show an average GF of 4.46 and 2.03, respectively.

Comparing the gauge factors in Table 1, it is clear that those of the 3D-printed samples are lower than that of the relevant filaments. It is important to point out, however, that the self-monitoring performance and the mechanical behavior of the filaments and 3D-printed samples cannot be directly compared, as the mechanism of electrical current flow is deeply different in the two cases and the 3D-printed samples mechanically behave as a multilayer structure. In fact, while the electrical resistance variation of the single filaments directly referrs to its composition and microstructure through the variation of CNT distribution within the matrix, in the 3D-printed samples, overall $\;\Delta R/R_{0}$ is the result of different concurring mechanisms as the current may flow through different paths.

**Figure 5:**
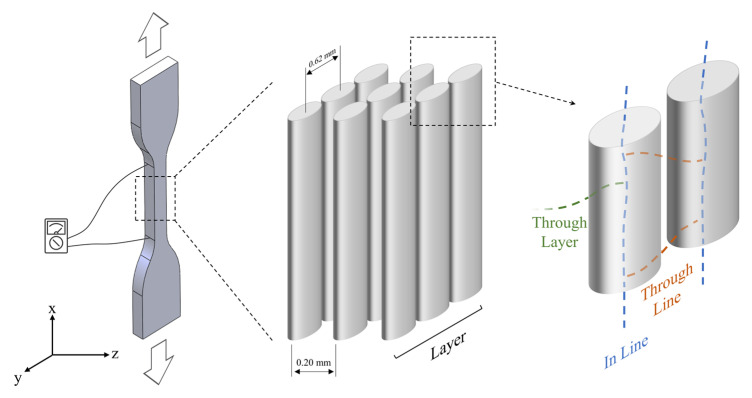
Schematic of the 3D-printed tensile specimens’ morphology and electrical conduction pathways.

Figure 5 proposes a schematic of possible current pathways within the 3D-printed sample, which are made of a stack of layers, with each layer composed by parallel conductive filaments parallel to the sample longitudinal axis. This morphology is due to the specific 3D-printing parameters used, namely 100% infill with a 0° raster angle. It has to be noted that different filament depositions lead to a different morphology and, consequently, different mechanical and electrical conductivity performances [22,36,70]. In our case, three major conduction pathways are available in the 3D-printed samples: (i) *in-line* conductivity along the length of the extruded filaments; (ii) *through-line* conductivity occurring within each layer between adjacent extruded filaments; and (iii) *through-layer* conductivity, with electron flow between the adjacent layers (Figure 5).

According to the proposed schematics, mechanical strain affects the three conduction pathways in different ways. *In-line* conductivity, with pathways running in continuous material, will be mainly affected by CNT redistribution and separation and is not dependent on the 3D-printing process. On the other hand, the presence of porosity and line–line separation will reduce the *through-line* conductivity, while delamination of the adjacent layers will impact the *through-layer* conductivity. The first mechanism is expected to play a major role in piezo-resistive behavior and to be responsible for the global $\;\Delta R/R_{0}$ vs. strain trend, while delamination and line–line separation cause local oscillations or step-like increases in electrical resistance, which are not accompanied by any event on the mechanical curve (i.e., Figure 4a at 2.2% and 4.7% strain).

Both *through-line* and *through-layer* conductivity are strictly dependent on the 3D-printing process. Indeed, during material deposition through the nozzle, the bonding process between adjacent lines and layers is driven by residual thermal energy in the material, which yields polymer chain diffusion across the interface [71]. Nozzle, bed and chamber temperatures, together with layer height and printing speed, are key factors in governing the extrusion and bonding process [72]. It is therefore clear how a shorter time interval between the deposition of two adjacent lines, compared to the interval between adjacent layers, involves greater residual thermal energy [11], and therefore determines a higher line-to-line bond strength compared to the poorer layer-to-layer bond. It is reported in the literature that inter-layer voids and adhesion greatly affect in-plane electrical conductivity, both in direct and alternating currents, while *through-line* conduction is affected to a lesser extent [73]. Even though the latter two conduction modes pose higher resistance to current flow, they still contribute to the overall equivalent resistance measured during the self-monitoring tests. If delamination or line debonding occur due to mechanical stress, the sudden interruption of the conduction pathways determines a jump in the measured electrical resistance variation. On the other hand, the interruption of those interfaces would not affect the mechanical response of the material because the interfaces are orthogonal to the applied stress and do not bear any load.

It can be concluded that delamination of 3D-printed layers produces the same effects on self-monitoring performance as those observed during the delamination of self-monitoring fiber-reinforced composite laminates [66]. The reported laminate-like behavior offers a possibility to detect damages in the direction orthogonal to the applied load and to increase the sensitivity of the composite material to the applied strain.

**Figure 6:**
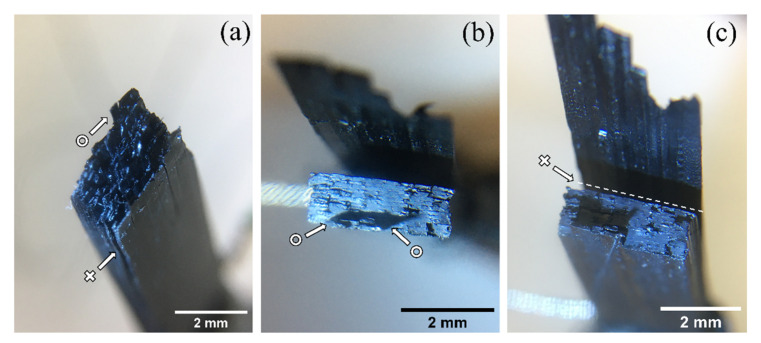
Optical microscopy on fracture surfaces of two different PEEK 3CNT 3D-printed samples ((**a**) sample 1, and (**b**,**c**) sample 2) showing line–line delamination (circle) and layer–layer delamination (cross).

The optical observation of the PEEK 3CNT 3D specimens after the tensile test (Figure 6) yields a better understanding of its mechanical and self-monitoring behavior (Figure 4a).

The presence of layer–layer delamination (highlighted with crosses) and line–line delamination within a single layer (highlighted with circles) is highlighted in the figure. As expected, the micrographs evidence that layer–layer delamination is more prevalent than line–line separation because, in the former, both polymer adhesion and inter-diffusion are weaker, according to the aforementioned temperature-driven bonding process. The extensive delamination of Figure 6b,c, in fact, occurs between the first and second printed layer, where the thermal mismatch is maximum. In fact, the first layer is deposited onto the printing plate and actively heated at 130 °C, causing the material to undergo rapid cooling. The mismatch causes reduced interlayer adhesion [74] and possible differential thermal contraction, which facilitate delamination during tensile testing.

The cyclic tests carried out on PEEK 3CNT (Figure 4d) highlight valuable and interesting self-monitoring behavior. The electrical resistance variation, in fact, is able to follow the mechanical stress and strain up to the final fracture, with no recorded electrical delay. Unlike some cases in the literature [75,76], no double or asymmetric peaks were recorded, highlighting a monotonic electrical response to mechanical strain. Comparing the maximum $\Delta R/R_{0}$ value recorded in each cycle with the values measured during monotonic testing (see Figure 4a) at the same tensile strain, it is possible to observe that the two values are perfectly comparable, as shown in the inset of Figure 4d. This behavior highlights a promising reliability of the system as a self-monitoring component also in cyclic loading conditions, as the maximum resistance value measured appears to be dependent only on the applied stress/strain. On the other hand, the $\;\Delta R/R_{0}$ value in the unloaded state (i.e., the valley of each cycle) appears to increase with each loading cycle. This behavior highlights a residual resistance variation which is ascribable to irreversible electrical path changes in the material, such as the rearrangement of the carbon nanotubes, cracks or local plastic deformation [77]. Finally, as strain gets closer to failure and delamination and cracks increase within the material, the $\;\Delta R/R_{0}$ curve tends to lose linearity and become noisy until fracture, in accordance with the quasi-static tensile testing results.

## 4. Conclusions

Self-monitoring PEEK–CNT nanocomposites at different filler loading levels (3, 5 and 10% by weight) have been extruded in filaments and 3D-printed via the FFF process. All the filaments show piezo-resistive behavior with an increase in electrical resistance when subjected to tensile stress, with a far higher sensitivity with respect to the typically employed strain gauges. These composite materials can be easily produced allowing for industrial-scale filament productions. Only 3 and 5 wt% formulations turned out to be 3D-printable due to the excessive brittleness of 10 wt% loaded filaments. All the 3D-printed samples showed self-monitoring behavior with decreased sensitivity with respect to the filaments, mainly due to the infill pattern and printing defects, which affect electrical conduction pathways. Three-dimensional-printed PEEK 3CNT samples that presented higher sensitivity were subjected to cyclic test, showing promising features for their use in health-monitoring structures.

## Figures and Tables

**Table 1:** Gauge factor for filaments and 3D-printed samples (with sample standard deviation).

	Gauge Factor	
	Filaments	3D-Printed Samples
PEEK Neat	n.a.	n.a.
PEEK 3CNT	14.5 (±0.9)	4.46 (±0.43)
PEEK 5CNT	3.36 (±0.31)	2.03 (±0.29)
PEEK 10CNT	1.99 (±0.24)	n.a.

n.a.—not available.

## Data Availability

The data presented in this study are available on request from the corresponding author.
